# Three cases of pediatric patients with testicular microlithiasis showing gynecomastia and testicular enlargement

**DOI:** 10.1186/1687-9856-2013-S1-P67

**Published:** 2013-10-03

**Authors:** Chong Kun Cheon, Su Yung Kim

**Affiliations:** 1 Department of Pediatrics, Children’s Hospital, Pusan National University, Beomeo-ri, Mulgeum-eup, Yangsan-si, Gyeongnam, 626-770, Korea

## Background

Testicular microlithiasis (TM) is a rare pathology characterized by localized or diffuse intratesticular foci of calcification. Its incidence in the pediatric population ranges from 1.1% to 4.2%.

The aetiology and the natural course of incidentally detected TM remain unclear.

## Objective

To report three cases of TM in boys who complained of gynecomastia and bilateral testicular enlargement.

## Results

Case 1) A 9.4-year-old boy presented with bilateral testicular enlargement accompanied by other pubertal signs. His bone age was 11 years and serum levels of LH and FSH after GnRH stimulation were within pubertal limits. Scrotal ultrasonography (US) showed TM in both testes. He revealed early puberty and no malignant evolution. Case 2) A 9.4-year-old boy with Down syndrome presented with bilateral testicular enlargement and accelerated growth velocity. His bone age was 11 years and serum levels of LH and FSH after GnRH stimulation were within pubertal limits. He had TM at US in both testes. He underwent right orchiopexy. He also revealed early puberty and no malignant change. Case 3) A 11-year-old boy complained of gynecomastia without other findings of puberty. His bone age was 11 years. He also had TM at US in both testes. No focal testicular lesion or malignancy developed during the review period.

## Conclusions

Our report underline the usefulness of scrotal US for finding an occult TM in a patient with gynecomastia without other findings of puberty. In addition, TM may be predictive factor for early puberty evolution, but a large number of patients with a longer follow-up period may be needed to accurately discern the possible association between TM and precocious puberty.

